# Water-soluble phenolic compounds produced from extractive ammonia pretreatment exerted binary inhibitory effects on yeast fermentation using synthetic hydrolysate

**DOI:** 10.1371/journal.pone.0194012

**Published:** 2018-03-15

**Authors:** Saisi Xue, A. Daniel Jones, Leonardo Sousa, Jeff Piotrowski, Mingjie Jin, Cory Sarks, Bruce E. Dale, Venkatesh Balan

**Affiliations:** 1 Biomass Conversion Research Lab (BCRL), Department of Chemical Engineering and Materials Science, Michigan State University, 3815 Technology Boulevard, Lansing, MI, United States of America; 2 DOE Great Lakes Bioenergy Research Center, Michigan State University, East Lansing, MI, United States of America; 3 Department of Biochemistry and Molecular Biology, Michigan State University, East Lansing, MI, United States of America; 4 Department of Chemistry, Michigan State University, East Lansing, MI, United States of America; 5 DOE Great Lakes Bioenergy Research Center, University of Wisconsin-Madison, Madison, WI, United States of America; 6 Department of Biochemistry, University of Wisconsin-Madison, Madison, WI, United States of America; 7 School of Environmental and Biological Engineering, Nanjing University of Science and Technology, Nanjing, China; 8 Department of Engineering Technology, Biotechnology Division, School of Technology, University of Houston, Houston, TX, United States of America; Tallinn University of Technology, ESTONIA

## Abstract

Biochemical conversion of lignocellulosic biomass to liquid fuels requires pretreatment and enzymatic hydrolysis of the biomass to produce fermentable sugars. Degradation products produced during thermochemical pretreatment, however, inhibit the microbes with regard to both ethanol yield and cell growth. In this work, we used synthetic hydrolysates (SynH) to study the inhibition of yeast fermentation by water-soluble components (WSC) isolated from lignin streams obtained after extractive ammonia pretreatment (EA). We found that SynH with 20g/L WSC mimics real hydrolysate in cell growth, sugar consumption and ethanol production. However, a long lag phase was observed in the first 48 h of fermentation of SynH, which is not observed during fermentation with the crude extraction mixture. Ethyl acetate extraction was conducted to separate phenolic compounds from other water-soluble components. These phenolic compounds play a key inhibitory role during ethanol fermentation. The most abundant compounds were identified by Liquid Chromatography followed by Mass Spectrometry (LC-MS) and Gas Chromatography followed by Mass Spectrometry (GC-MS), including coumaroyl amide, feruloyl amide and coumaroyl glycerol. Chemical genomics profiling was employed to fingerprint the gene deletion response of yeast to different groups of inhibitors in WSC and AFEX-Pretreated Corn Stover Hydrolysate (ACSH). The sensitive/resistant genes cluster patterns for different fermentation media revealed their similarities and differences with regard to degradation compounds.

## Introduction

In the fossil fuel-based economy, crude oil is the primary feedstock source for producing transportation fuels and industrial chemicals. Dependence on crude oil causes energy security concerns and greenhouse gas emissions drive climate change. These forces have triggered worldwide research towards the development of alternative, sustainable sources of energy [1]. A renewable alternative to fossil fuel-derived liquid fuels, such as gasoline and diesel, is lignocellulosic biofuels. These are expected to play a major role in satisfying our energy needs [2,3]. Unlike corn grain-based ethanol, where the starch can be readily hydrolyzed to fermentable sugars using enzymes, the lignocellulosic biomass used in second-generation biofuels has naturally evolved to be highly recalcitrant to enzymatic deconstruction by fungi and bacteria [4]. Therefore, pretreatment of lignocellulosic biomass is necessary for biofuel production by reducing the recalcitrance of biomass and enabling efficient conversion to monomeric sugars [5]. Pretreatment processes are commonly performed under high temperature, high pressure, caustic, or acidic conditions, which generate degradation compounds that inhibit microorganisms [6]. Under acidic conditions, carbohydrates present in the biomass degrade into furfural or hydroxymethylfurfural, and the lignin degrades into a variety of phenolic compounds [7]. In contrast, the Ammonia Fiber Expansion (AFEX^TM^) process produces many ammoniated compounds, which are significantly less inhibitory than their acid counterparts [8,9]. A previous comparison of AFEX and dilute acid treated corn stover showed that dilute acid pretreatment produces 316% more acidic compounds, 142% more aromatics, and 3555% more furans than AFEX, but no nitrogenous compounds [8].

Notwithstanding the less toxic degradation products generated, the sugar utilization efficiency of ethanol production using ammonia-pretreated biomass still requires improvement. One major issue is the low xylose consumption rate during hexose/pentose co-fermentation, which largely results from pretreatment-derived biomass decomposition products, ethanol, and other fermentation metabolites [9–12]. Thus, novel pretreatment technologies that further reduce toxic degradation products content in biomass were needed to minimize xylose utilization problems faced during yeast fermentation. Extractive-Ammonia (EA) is a newly developed pretreatment technology that selectively extracts lignin present in biomass. Compared to AFEX, EA uses higher ammonia-to-biomass loading and lower water loading, generates a separate lignin stream to extract up to 45% of the lignin from lignocellulosic, and removes most of the degradation products [13]. Thus, EA-pretreated corn stover was found to have reduced lignin and degradation product content, enhanced digestibility of cellulose due to formation of cellulose III, and improved hydrolysate fermentability [13].

To study the inhibitory effects of degradation compounds, the DOE Great Lakes Bioenergy Research Center (GLBRC) has formulated a chemically-defined SynH to mimic real AFEX corn stover hydrolysate (ACSH) [9]. Synthesized aromatic compounds were added into the control media based on ACSH composition analysis to better understand the complex inhibitory effects [9–11, 14, 15]. This artificial media has been proven helpful in testing the inhibitory effects of degradation compounds on engineered microbial strains. The aromatic compounds used in those studies, however, cannot fully represent the real ACSH due to the incomplete analysis of compounds present in ACSH, and different conformational isomers of synthesized compounds. Therefore, we explored an alternative method of producing SynH using naturally-derived compounds to better represent the inhibitory effects in ACSH. Also, microbial fermentation is done in aqueous media, so different water-soluble organic compounds and their relative concentrations will interact with microbes and influence their performance and viability.

In this study, water soluble components (WSC) were separated from crude lignin streams produced during EA pretreatment. These soluble compounds were added to base media to mimic real inhibitors in ACSH that affect microbial fermentation. Water extraction followed by ethyl acetate extraction were conducted successively to separate the WSC, especially the phenolic nitrogenous compounds in crude lignin stream obtained after EA pretreatment. Inhibition effect for WSC on yeast fermentation using SynH were observed. New inhibitors that were different from those known to be present in ACSH were identified using High Performance Liquid Chromatography (HPLC) and Liquid Chromatography-Mass Spectrometry (LC/GC-MS). Chemical genomics profiling was performed to further understand the gene response profile and metabolic pathways that are being inhibited by WSC in SynH. Gene deletion studies could help guide genetic engineering of yeasts to overcome inhibitory effects of lignocellulosic hydrolysates.

## Materials and methods

### Biomass

Corn stover used in this study was produced from corn (Pioneer 36H56) planted and harvested during 2010 at the Arlington Agricultural Research Station (Columbia County, WI). No specific permissions were required for these locations/activities as this belong to university of Wisconsin agriculture research station. Also, the field studies did not involve endangered or protected species.

### Chemicals

All chemicals and reference standards used for composition analysis were purchased from Sigma–Aldrich (St. Louis, MO, USA) if not mentioned otherwise.

### Biomass and AFEX pretreatment

Corn stover used in this study was produced from corn (Pioneer 36H56) planted and harvested during 2010 at the Arlington Agricultural Research Station (Columbia County, WI). The corn stover was pretreated using the AFEX process as previously described [16] using a 5-gallon reactor, under the condition of 1:1 ammonia to biomass ratio, 60% moisture, 100 °C, and 30 minutes’ reaction time. Composition analysis, following the method of Sluiter et al., [17, 18] gave glucan, xylan, acid insoluble lignin, and ash contents of 31.4%, 18.6%, 13.08%, and 13.39%, respectively. The pretreated corn stover was stored at 4 °C.

### Obtaining the crude lignin stream from EA pretreatment

A crude lignin stream was separated and collected following EA pretreatment of corn stover. EA pretreatment was applied to corn stover as previously described in the literature [13] using 6:1 ammonia-to-biomass weight ratio (NH_3_:BM) for 30 minutes at 120°C. EA pretreatment removed 16 percent (wt%) of the biomass or about 44 wt% of the total lignin present in the biomass. This extracted lignin stream was used as the starting material for subsequent water extraction and ethyl acetate extraction. The composition of the lignin stream was reported previously [13].

### Water extraction of crude lignin obtained after EA pretreatment of corn stover

WSC were extracted from the crude lignin stream produced during EA pretreatment process. Distilled water was added and vortex-mixed with the lignin stream in a 10:1 (volume to mass) ratio. Water extraction was conducted in shake flask by incubating the samples in an incubator maintained at 50 °C for 2 h at 250 rpm. After the 2 h washing and extraction, the slurry was cooled to 4°C, and was then centrifuged and filtered through glass fiber filter (pore size 10 um). The filtrate was collected as WSC and lyophilized using a freeze dryer (Free Zone plus 6 Liter Cascade Console Freeze Dry System, LABCONCO). The WSC was further used for composition analysis, ethyl acetate extraction, and yeast fermentation.

### Ethyl acetate extraction of WSC

**Fig 1:**
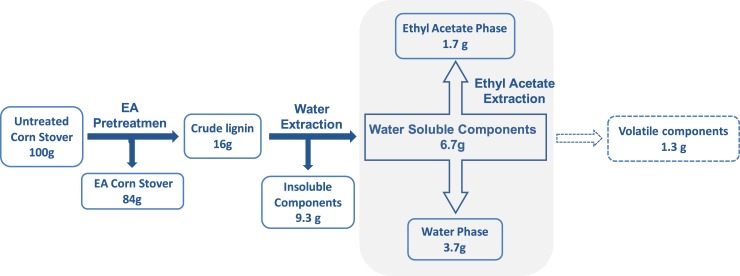
Methodology and mass balance of water extraction and ethyl acetate extraction of crude lignin.

Ethyl acetate extraction was performed to separate and enrich the phenolic compounds from WSC. The pH of WSC supernatant (before lyophilizing) was adjusted between pH 2 to pH 3 to protonate the phenolic acids and other aromatic components of the mixture, and increase their solubility in ethyl acetate. Ethyl acetate and the supernatant (1:1 volume ratio) were added into the separation funnel. The liquid was fully mixed in the funnel for 10 minutes and allowed to sit for 30 minutes for phase separation. The bottom water phase was collected and centrifuged at 4,400 rpm for 5 minutes. The top ethyl acetate phase, enriched with phenolic compounds, was collected in a separate bottle. The same volume of ethyl acetate was added again to the water fraction, and the extraction procedure was repeated twice more. After three rounds of ethyl acetate extraction, all the ethyl acetate fractions were combined and dried using a rotary evaporator system (BUCHI Rota vapor R-210 and Heating Bath B-491) to concentrate the phenolic compounds. The liquid was concentrated until 5 to 10 mL liquid was left in the round bottom flask. This ethyl acetate fraction was air-dried in room temperature to avoid overheating the phenolic compounds. The remaining water fractions in the separation funnel was dried using the rotary evaporator for mass balance and other microbial inhibition studies, and were further concentrated using a freeze drier connected to an organic trap (Fig 1).

### Fractionation of WSC using size exclusion chromatography (SEC)

SEC was performed to fractionate WSC based on the molecular weights. A GE Healthcare XK column (150 mL bed volume, Tube Height: 400 mm, Column internal diameter: 16 mm) was packed with Bio-Rad P6 gel (Polyacrylamide, 1-6kDA). Water was used as the mobile phase with 1mL/min flow rate. About 0.70 mL of WSC was injected for each run. A total of 96 fractions (A1-H12) were collected in tubes using an automatic sample collector. Fractions with higher molecular weight would come out first in row A, followed by row B to row H.

### Composition analysis of sugars and WSC

Sugars and organic acids in the WSC and hydrolysate were analyzed using an HPLC equipped with a Bio-Rad (Hercules, CA) Aminex HPX-87P column and de-ashing guard column. Column temperature was held at 80 °C and 5mM H_2_SO_4_ in water flowing at 0.6 mL/min was used as the mobile phase.

LC-MS analyses of WSC were conducted using a Waters LCT Premier mass spectrometer equipped with Shimadzu LC-20AD ternary pumps and controller with high pressure mixer, Shimadzu column oven and CTC PAL auto-sampler, and operated using electrospray ionization. A Supelco Ascentis Express C18 column (2.1 x 50 mm, 2.7 μm particles) was used, with column temperature held at 55 °C. Separations were conducted using gradient based on solvent A: aqueous formic acid and solvent B: methanol. The gradient as set to vary between 99% (0.50 min hold) to 0 in 10 minutes, held for 4 minutes, and set back to 99% for 1 minute.

GC-MS was conducted using a 30 meter-long VF5MS column plus a 10 meter-long EZ-Guard guard column, on a Waters GCT Premier mass spectrometer coupled to an Agilent 6890 GC with auto-sampler. Data acquisition employed 70 eV electron ionization, with data acquisition performed over *m/z* 40–600 at 0.2 seconds/spectrum (with dynamic range extension). The temperature program was set to vary between 40 °C (2 minutes hold), 6°/min to 300°, and held for 5 minutes. Helium was used as carrier gas with a flow rate of 1.3 mL/min. Compounds identified using GC-MS used electron ionization spectra in the NIST11 mass spectrum library.

Free amino acid analysis was outsourced to the Molecular Structure Facility, UC Davis, using a Hitachi L-8900 Amino Acid Analyzer. Heavy metal analysis was also outsourced and analyzed by the UW Soil & Plant Analysis Lab using standard protocols. The Standard Operation Procedures of ICP-OES/MS are available at this website (http://uwlab.soils.wisc.edu/ files/procedures/ICPMS.pdf).

### High solid loading enzymatic hydrolysis

The AFEX-pretreated corn stover samples were hydrolyzed at 6% glucan loading in a fermenter equipped with a pitched blade impeller. Hydrolysis was performed over a period of 3 days with 20 mg protein/g glucan enzyme loading at 50 °C and 1,000 rpm. Enzymatic hydrolysis was performed using a commercial enzymes mixture including Cellic® Ctec2 10 mg protein/g glucan (in pretreated biomass), Htec2 (Novozymes, Franklinton, NC), 5 mg protein/g glucan and Multifect Pectinase (Genencor Inc, USA), 5 mg protein/g glucan. Samples were taken every 24 h. The hydrolysate was harvested by centrifugation at 6000 rpm for 30 min and then 14,000 rpm for 30 min to remove unhydrolysed solids. Hydrolysate was then sterile filtered through a 0.22-um filter cup. The filtered hydrolysate was stored at 4 °C in a sterile bottle prior to charcoal fractionation (described below). Samples obtained from compositional analysis were subjected to HPLC using Bio-Rad Aminex HPX-87H column to determine sugar concentrations as described below. After 3 days of hydrolysis (i.e., 72 h), the overall mass balances for the pretreated solids were determined using NREL protocol as described previously [14].

### Synthetic hydrolysate (SynH) preparation

The formulation of SynH is based on the composition of ACSH produced in the enzymatic hydrolysis, with glucose concentration of 60 g/L and xylose concentration of 30 g/L. The development of the SynH recipe is described elsewhere [15, 19, 20]. Carbohydrates, nitrogenous compounds, vitamins, minerals, and other necessary nutrients were added into distilled water as base media. Detailed composition information of the updated SynH used in this work was listed in the S2 Table.

### Microorganism and seed culture preparation

*Saccharomyces cerevisiae* GLBRC-Y128 was used for this study, having previously been genetically modified and adapted for xylose utilization [21,22]. Xylose isomerase and xylulokinase genes were introduced to facilitate xylose utilization. Seed cultures were prepared from glycerol stocks stored at -80 °C. Seed culture media contained 100 g/L dextrose, 25 g/L xylose, 20 g/L tryptone, and 10 g/L yeast extract. Erlenmeyer flasks (250 mL) containing 100 mL of seed culture media were inoculated with 0.1 OD_600_. The cultures were incubated at 30 °C and 150 RPM in shaker incubators under micro-aerobic conditions for 22 h before inoculation in SynH. Measurements of cell population was conducted by measuring the optical density at 600 nm to measure the cell concentration of the fermentation broths. The fermentation broth was diluted and re-suspended to the detection range of 0.1–1 OD_600_, and calibrated for original cell concentration.

### Chemical genomic profiling of ACSH and WSC

Chemical genomic analysis of hydrolysate and WSC was performed as described previously using a collection of ~4000 yeast deletion mutants [14,23]. About 200 μL cultures with the pooled collection of *S*. *cerevisiae* deletion mutants were grown in the different SynH or yeast extract (10 g/L), peptone (20 g/L), and dextrose (20 g/L) (YPD) medium in triplicate for 48 h at 30 °C under aerobic conditions. Genomic DNA was extracted from the cells and mutant-specific molecular barcodes were amplified using specially designed multiplex primers as described previously [24,25]. The barcodes were sequenced using an Illumina HiSeq2500 in rapid run mode (Illumina, Inc., San Diego, CA). The average barcode counts for each yeast deletion mutant in the replicate hydrolysates were normalized against the YPD control to define sensitivity or resistance of individual strains (chemical genetic interaction score). Strains with low read counts were omitted from analysis. A resistant mutant has a positive interaction score, whereas a negative score indicates a sensitive mutant. The pattern of genetic interaction scores for all mutant strains represents the chemical genomic profile or ‘‘biological fingerprint” of a sample [23–25]. Correlations of the chemical genomic profiles across cycles were calculated using Spot-fire 5.5.0 (Tibco, Boston, MA, USA). The cluster-gram of the chemical genomic profiles were created in Cluster 3.0 [26], and visualized in Tree-view (v1.1.6r4) [27]. A Bonferroni-corrected hypergeometric distribution test was used to search for significant enrichment of GO terms among the top 10 sensitive deletion mutants [28].

## Results and discussion

### Mass balance of water extraction and ethyl acetate extraction of lignin streams

As mentioned previously, EA pretreated corn stover yielded more fermentable sugars compared to the AFEX process with a 60% lower enzyme loading [13]. Removing water-soluble, lignin-derived inhibitors is expected to greatly relieve their inhibitory effects on microbial fermentation [15,20]. To isolate the key inhibitors that affect ethanol productivity, we performed water extraction to separate the WSC from the EA-derived crude lignin stream. We also subjected the soluble components to liquid-liquid partitioning between ethyl acetate and water to selectively enrich water-soluble aromatic compounds (Fig 1). A mass balance for water extraction and ethyl acetate extraction is shown in Fig 1. About 40 wt% of crude lignin stream was water-soluble. The WSC were subjected to ethyl acetate extraction to enrich phenolic compounds, and this fraction was used in microbial fermentation and composition analysis. A mass balance with 80 wt% total recovery was achieved following ethyl acetate-water partitioning. About 26% of WSC were extracted in the ethyl acetate phase, mostly phenolic compounds. Some of the phenolic compounds could not be recovered during vacuum drying of ethyl acetate fractions due to their volatile nature. About 55 wt% of WSC remained in the water phase after ethyl acetate-water partitioning. To identify major components in WSC and the ethyl acetate fractions, composition analyses including SEC, HPLC, LC-MS and GC-MS, were conducted.

### Fractionation and composition analysis of WSC

**Fig 2:**
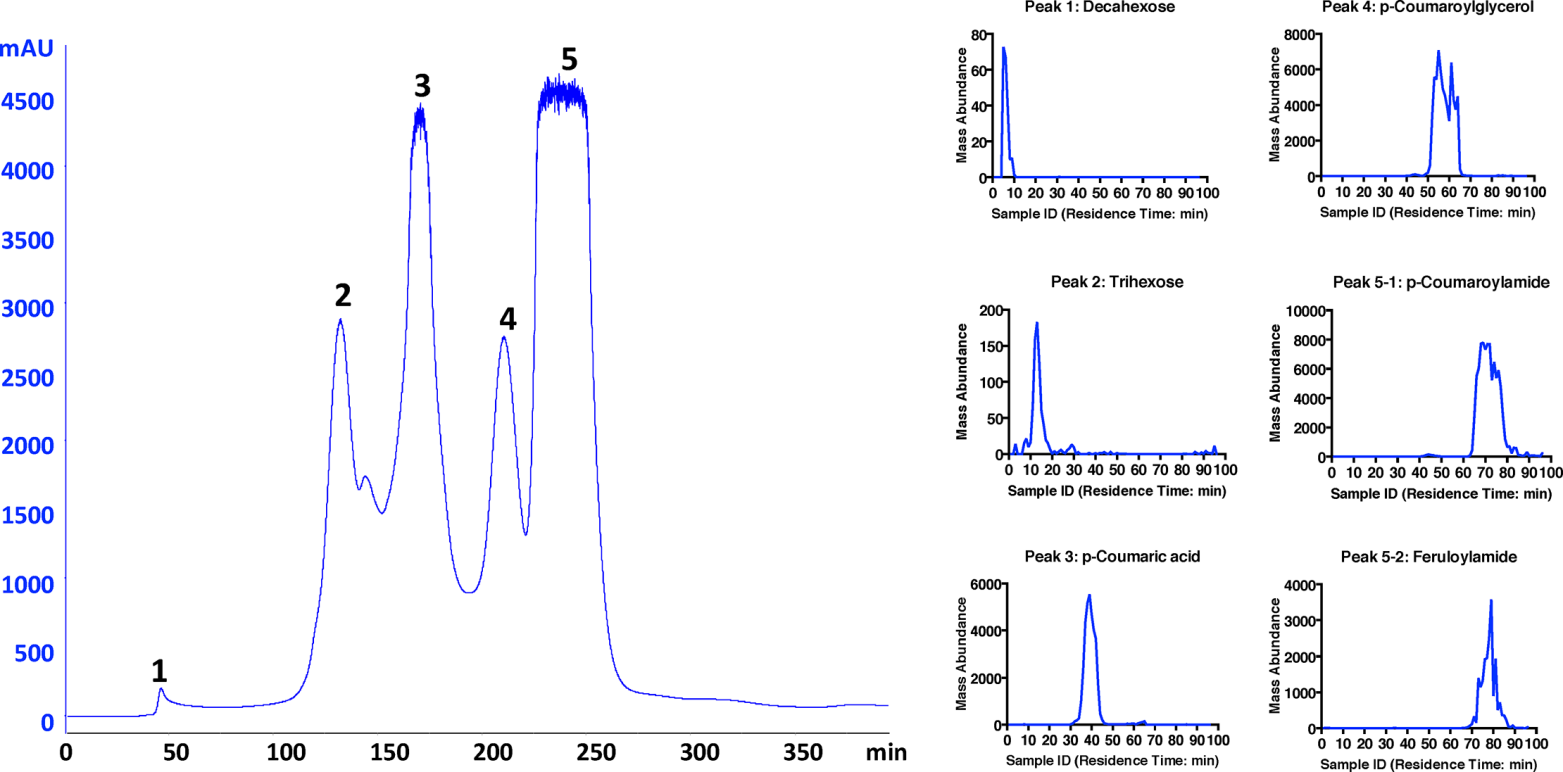
Size exclusion chromatography (SEC) fractionation and liquid chromatography-mass spectrometry (LC-MS) screening of WSC. Here, (A) SEC of WSC using P6 gel (Polyacrylamide, 1–6 kDa, water as mobile phase) with UV-Vis detector at 280nm. 96 SEC fractions from A1-H12 were collected in a 96-well microtiter plate and (B) LC-MS screening of the fractions collected in SEC were performed in the same order as they were collected (A1-H12). Peak 1 to peak 5: Measured signals from LC-MS extracted ion chromatograms for abundant components profiled by LC-MS and assigned based on SEC retention times, aligned from peak No. 1 to peak No. 5 in the SEC chromatogram.

To obtain an estimation of the molecular-weight range of WSC components, we first fractionated the WSC through SEC column, which was packed with P6 gel (separation range 1–6 KDa). As shown in Fig 2A, five major UV-absorbing peaks were eluted. It was anticipated that higher molecular mass components would elute earliest. In Fig 2B, fractions collected in SEC were subjected to LC-MS screening in the same order as they were collected (from A1 to H12). At least one major compound was assigned to each of the five SEC peaks. Deca-hexose was detected in peak 1, followed by tri-hexose being detected in peak 2, showing that oligosaccharides were soluble components following EA processing [8, 29, 30]. Additional xylo-oligomers were detected in the first two peaks (data not shown). *p*-Coumaric acid and *p*-coumaroylglycerol were detected in SEC peaks 3 and 4 respectively. Both were major lignin degradation products in corn stover pretreatment. In peak 5, *p*-coumaroylamide and feruloylamide were detected. They are major ammoniated lignin degradation products produced by ammonia pretreatment ammonolysis of polyphenolic esters. It is worth noting that *p*-coumaroylamide corresponded to more than 80 wt% of total mass being detected by LC-MS, indicating that nitrogenous phenolic compounds were the most abundant and one of the most inhibitory components in WSC. The LC-MS screening also revealed that more than 98% of LC-MS peak area was explained by compounds smaller than 3 KDa, which was consistent with our assumption that most water-soluble inhibitors were small molecules.

**Fig 3:**
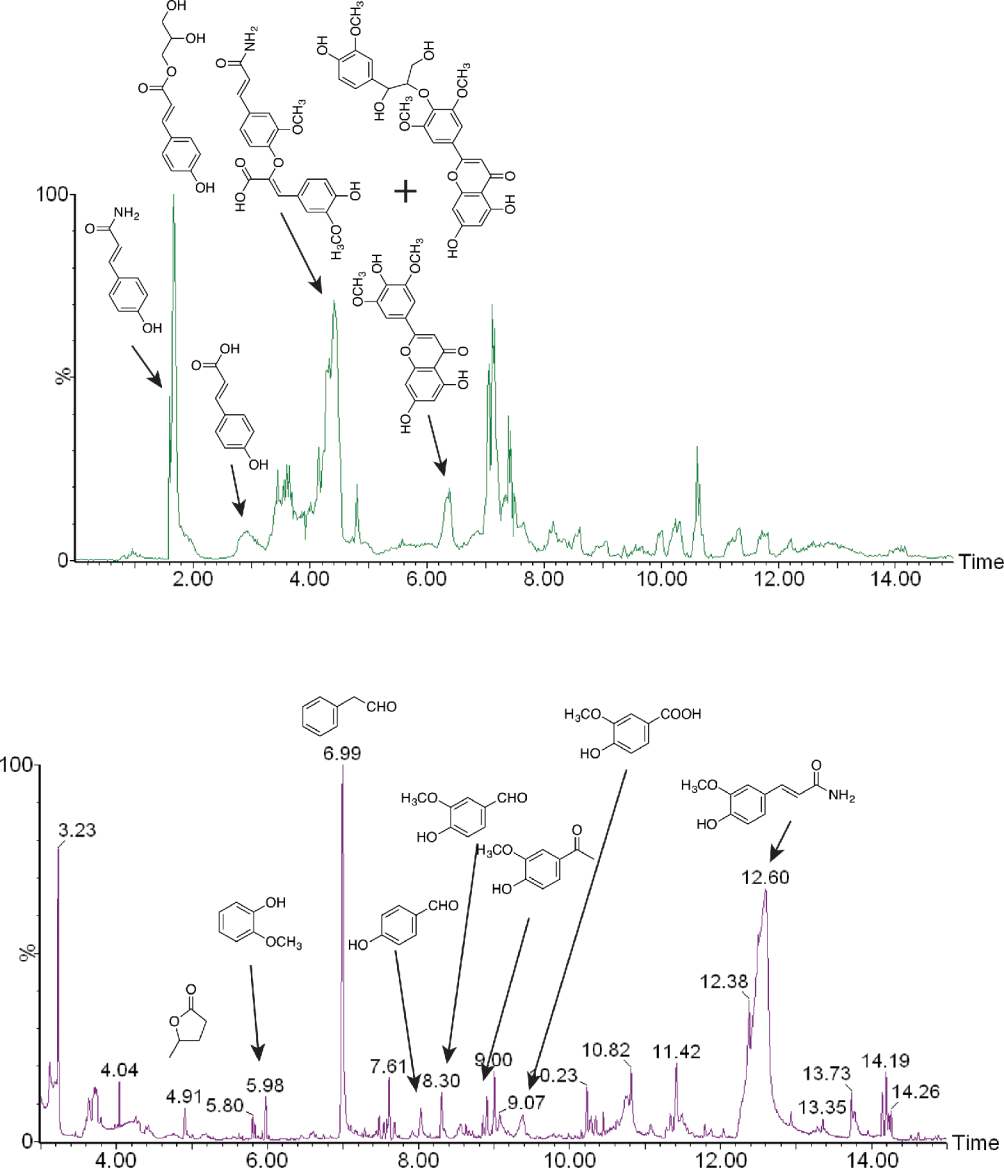
Water soluble aromatic compounds isolated from EA crude lignin stream using ethyl acetate. Here, (A) Ultra-high performance liquid chromatography-mass spectrometry (UHPLC-MS) and (B) gas chromatography-mass spectrometry (GC-MS) chromatograms were used to identify phenolic compounds.

As phenolic compounds were enriched in the organic phase after ethyl acetate-water partitioning, ultra-high performance liquid chromatography-mass spectrometry (UHPLC-MS) and GC-MS were employed to identify major components. In Fig 3A, phenolic acid, phenolic amides including diferulate amides, and lignin derivatives based on tricin and its conjugates with monolignols were identified. In addition, aromatic aldehydes/ketones, and furanones were identified from GC-MS analysis (Fig 3B), some of which were produced from Maillard reaction and of reducing sugars and ammonolysis of lignin during ammonia pretreatment [8, 31–34]. Maillard reaction products not only accounted for the brown color of ammonia-treated biomass and WSC, they also acted as inhibitors for microbial fermentation [8,32]. Identified phenolic and nitrogenous compounds also explained the reason why EA pretreated biomass had a much better performance in xylose fermentation and ethanol production by removing lignin streams.

The components remaining in the water phase after ethyl acetate-water partitioning were also analyzed. LC-MS analysis of water phase fraction showed no signal attributed to phenolic compounds, consistent with their greater solubility in ethyl acetate. Instead, monomeric sugars were identified, which was consistent with the result of LC-MS screening of WSC. On top of LC-MS, HPLC analysis of sugar and organic acids in both WSC and water phase after ethyl acetate extraction showed small amount of free monomeric sugars (Table A in S1 Table). Glycerol and acetate were also detected (Table B in S2 Table). Free amino acid analysis and heavy metal analysis are summarized in Table C and D in S1 Table. High nitrogen levels were identified compared with all other elements levels (Table D in S1 Table), which was consistent with the degradation product profiles after ammonia pretreatment of corn stover [8]. Due to the distinct composition profiles of components in the phenolic phase and the water phase after ethyl acetate-water partitioning, we tested their inhibitory effects on microbial fermentation performance, respectively.

### Using SynH to characterize the inhibitory effect of WSC on yeast fermentation

To investigate the inhibitory effect of WSC on microbial fermentation, a chemically-defined hydrolysate media with monomeric sugars and necessary nutrients was needed. Researchers at the GLBRC formulated a SynH media based on the composition analysis of 6% solids loading ACSH [8, 9], which was designed to mimic the composition and behavior of common biomass hydrolysate [5]. Table 1 show the SynH recipe which consists of six groups of components. Detailed concentration for each component can be found in S2 Table. Carbohydrates, nitrogenous compounds, vitamins and minerals provided carbon sources, nitrogen sources and other necessary nutrients as base media. Degradation products produced from pretreatment process, on the other hand, acted as inhibitory compounds. Two major groups of degradation products were carbohydrate derivatives (*e*.*g*. aliphatic acids, furans, acetamide, pyrazines, imidazoles) and lignin derivatives (aromatic acids and amides). The inhibitory degradation components could be replaced with other types of compounds to test their effect on microbes. In this work, we used WSC and constituents in its ethyl acetate extracts to investigate their inhibitory effects on yeast fermentation.

**Table 1:** The major compositions in SynH. Detailed recipe with concentrations can be found in S2 Table.

**Carbohydrates**	Monomeric sugars (glucose, xylose, arabinose, mannose, galactose, fucose)
**Nitrogenous compounds**	Ammonium chloride, 20 amino acids, nucleobases
**Vitamins**	Panthothenic acid, thiamine pyridoxine, etc.
**Mineral salts**	Zinc chloride, Manganese (II) chloride, etc.
**Carbohydrate degradation products/ plant metabolites**	Aliphatic acids, Furans, Acetamide, Pyrazines, Imidazoles
**Lignin degradation products**	Aromatic acids and amides

**Fig 4:**
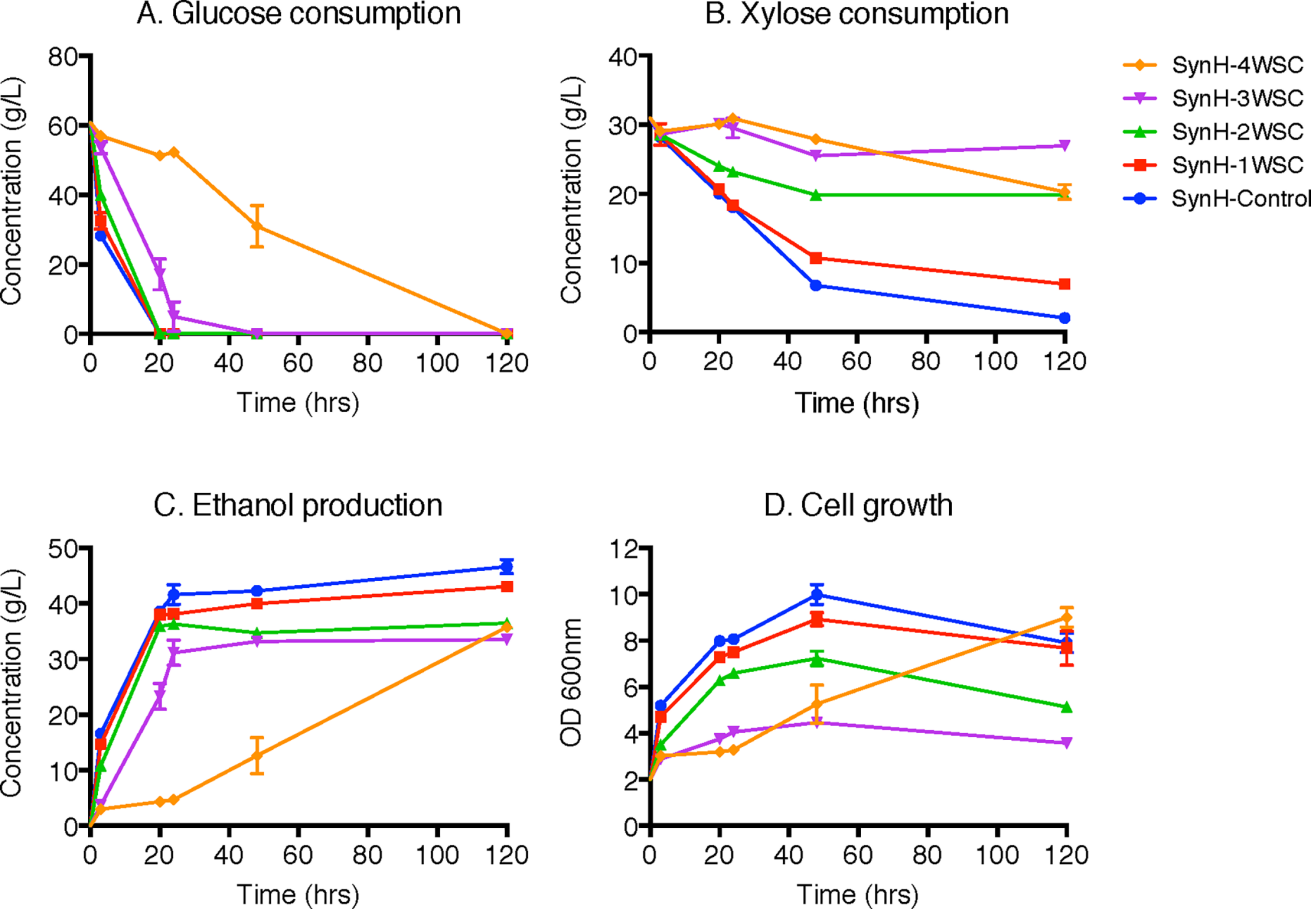
Fermentation performance of Y128 under varying concentrations of WSC. (A) Glucose consumption; (B), Xylose consumption; (C), Ethanol production and (D), Cell growth OD_600_. Syn-4WSC: SynH with 40 g/L WSC added; SynH-3WSC: SynH with 30 g/L WSC added; SynH 2WSC: SynH with 20 g/L WSC added; SynH-1WSC: SynH with 10 g/L WSC added; SynH-Control: SynH-base media with no inhibitors added. Fermentation was conducted in Erlenmeyer flasks (50 mL) at pH 4.8, 30 °C and 150 RPM with inoculum at 2 (OD_600_).

A haploid Y128 yeast strain was developed by GLRBC with the ability to rapidly co-ferment glucose and xylose anaerobically even in the presence of ACSH inhibitors [22]. The freeze-dried WSC was re-dissolved into SynH control media at varying concentrations. As shown in Fig 4, the concentration of WSC increased from 10 up to 40 g/L for SynH-1WSC to SynH-4WSC, respectively. Here, 1WSC represents 10 g/L WSC which were re-dissolved in SynH-base media. Fermentation was carried out in Erlenmeyer flasks (50 mL) at pH 4.8, 30 °C and 150 RPM with inoculum at 2 (OD_600_). Trends in glucose consumption and the first 48 h of xylose consumption as well as ethanol production demonstrated that the inhibitory effects of WSC increased as their concentration increased. Adding WSC resulted in a lag phase for glucose and xylose consumption during the first 24 h, especially at higher concentrations.

However, once the initial concentration of WSC reached 40 g/L, xylose consumption, ethanol yield and cell growth were no longer reduced after 48 h of fermentation. For example, the xylose concentration at 120 h for SynH-4WSC very close to SynH-2WSC (20.3 g/L versus 19.8 g/L) and much lower than SynH-3WSC (20.3 g/L versus 27.0 g/L). The ethanol concentration at 120 h for SynH-4WSC was higher than SynH-3WSC (35.8 g/L versus 33.5 g/L), and measurements of cell growth of SynH-4WSC in Fig 4 even showed a reversed effect. The OD_600_ reached 9.0 at 120 h, which is the highest level achieved in all the fermentation media tested here.

Since two different trends are observed here, an early stage lag phase versus late stage cell growth enhancement, at least two groups of components with distinct inhibitory effects must exist in WSC. In addition to fermentation with high initial inoculum (OD 2 (Fig 4)), we also tested the fermentation with low initial inoculum at OD 0.1. The yeast fermentation at different initial inoculums had similar trends in sugar utilization, ethanol production and cell growth. However, WSC had an amplified effect (both on early stage inhibition and late stage improvement) at low inoculum levels of OD 0.1 compared to high inoculum levels (OD 2), proving that a high inoculum at the beginning of fermentation could help with the microbial resistance to inhibitors in hydrolysate.

### Using SynH to characterize the binary inhibitory effects of different ethyl acetate fractions on yeast fermentation

**Fig 5:**
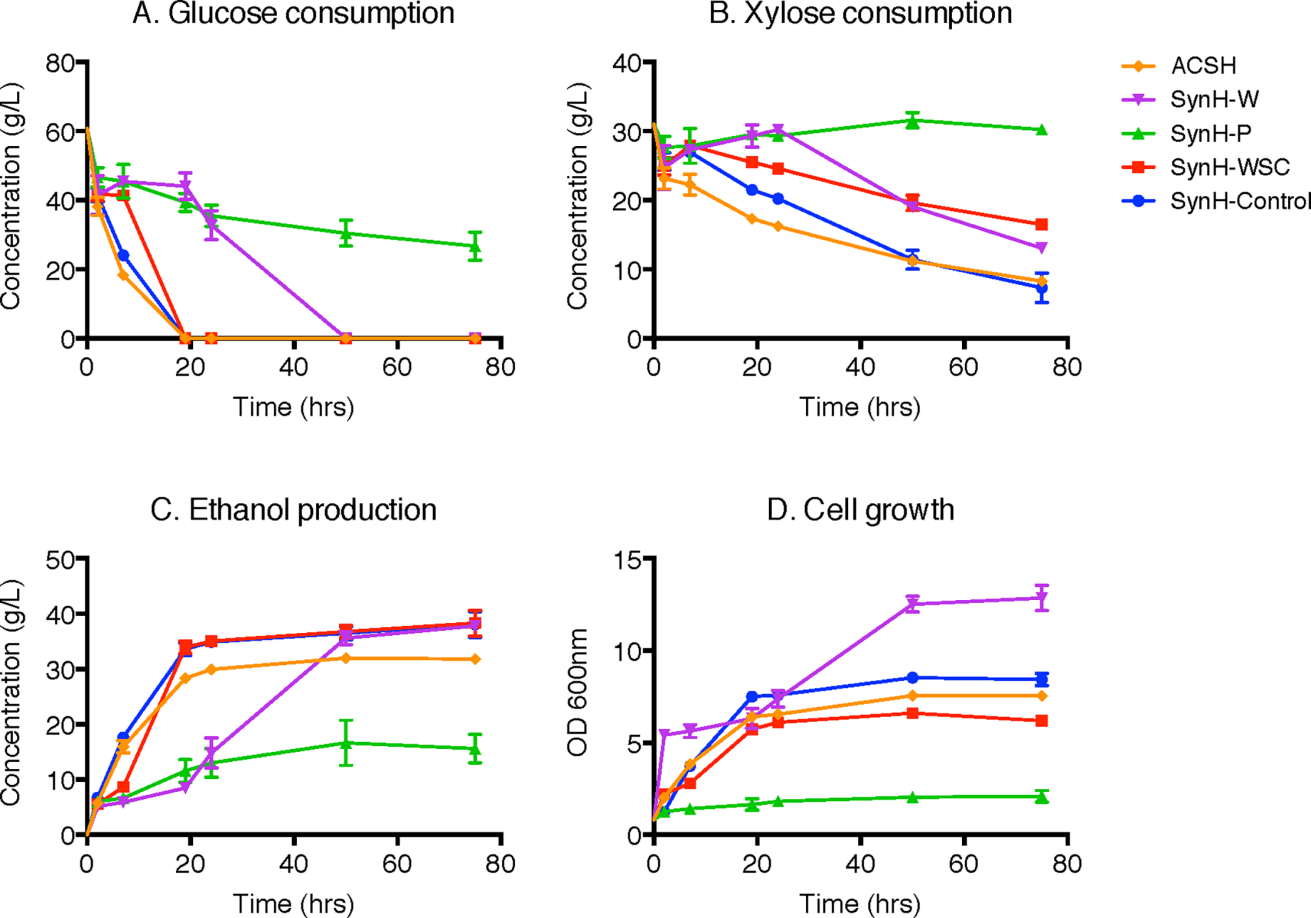
Fermentation performance of Y128 using different WSC fractions. **Here,** (A) Glucose consumption; (B) Xylose consumption; (C) Ethanol production and (D) Cell growth OD_600_. ACSH: AFEX corn stover hydrolysate; SynH-W: SynH with 20 g/L water phase extract after ethyl acetate-water partitioning; SynH-P: SynH with 20 g/L ethyl acetate phase extract after ethyl acetate-water partitioning; SynH-WSC: SynH with 20 g/L WSC; SynH-Control: SynH-base media with no inhibitors added. Both phenolic compounds and nutrient components were re-dissolved in SynH-base media at 20 g/L. Fermentations were conducted in Erlenmeyer flasks (50 mL at pH 4.8, 30 °C and 150 RPM with inoculum at 0.8 OD_600_.

To further investigate the complex dual effect of WSC on the yeast fermentation, we performed additional Y128 fermentations for 72 h using organic and aqueous phase extracts of WSC after ethyl acetate-water partitioning (Fig 5). As we found that SynH with 20 g/L WSC mimicked real ACSH in cell growth, sugar consumption and ethanol production, both SynH-2WSC and ACSH were used as positive controls in this group of experiments. SynH-control media was kept as the negative control. SynH-W represented the water phase extract after ethyl acetate extraction, which contains soluble sugars, liquids and other nutrients. SynH-P represented the organic phase extract after the ethyl acetate-water partitioning, in which phenolic compounds were enriched.

Both SynH-P compounds and SynH-W components were re-dissolved in SynH control media at 20 g/L. After separating ethyl acetate WSC extractives from the aqueous fraction, the binary behaviour observed in previous experiments (Fig 4) disappeared. The ethyl acetate fraction, enriched in phenolic compounds, exerted the strongest inhibitory effects on Y128 fermentation among all the media tested. Less than 50% of the glucose was consumed, and xylose was barely utilized. The ethanol yield after 72 h was 16 g/L, compared with ~38 g/L for all other media, and cell growth after 72 h was inhibited to OD 2.1, much lower than all other fermentation media tested.

In contrast, even though a short lag phase was observed for the first 24 h, SynH-W boosted the cell growth to OD 13 at 72 h, much higher than SynH-control media (OD 8.4) (Fig 5). The concentration of monomeric sugars alone in the nutrient fraction is too low to account for cell growth enhancement. Thus, we suggested that lipids, oligomeric sugars and potential cell protectants were major contributors to enhanced fermentation performance. Phenolic compounds and water-soluble nutrients together account for the dual inhibitory effect of WSC on ethanol fermentation.

### Chemical genomics fingerprints of WSC on yeast growth

**Fig 6:**
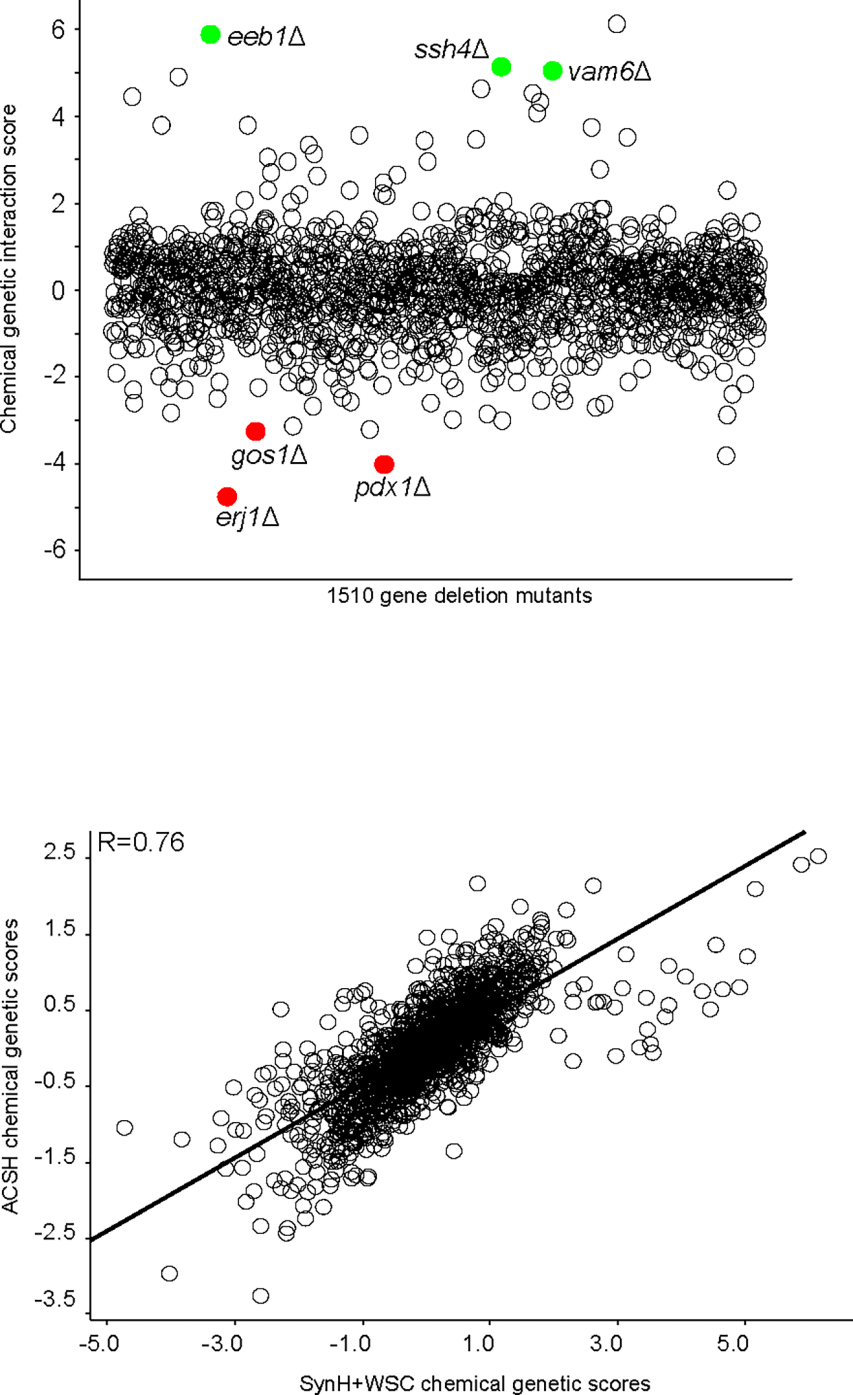
Chemical genomic profiling of WSC on using a yeast deletion strain library. (A). Chemical genomic profile of SynH+WSC, with the top sensitive deletion mutants (red) and top resistant mutants (green) highlighted (mean profile n = 3); (B). Gene clusters correlation between SynH, ACSH and SynH+WSC (n = 3). Mutants in *ERJ1* (involved in ER protein folding), *PDX1* (a subunit of the mitochondrial dehydrogenase complex), and *GOS1* (involved Golgi transport) were especially sensitive to WSC. The sensitive genes gave insight into the mechanism of toxicity, confirming that cell membranes were the likely target of WSC toxicity; and overexpression of the sensitive genes could be used to confer resistance. Comparing the chemical genetic profiles between different hydrolysates and fermentation media, we found that the profile of SynH + WSC exhibited a high correlation with that of ACSH (Fig 6B, R = 0.69), and showed greater similarity to ACSH compared to SynH (Fig 7). The strong correlation suggests that the degradation compounds in WSC can represent the real inhibitors in ACSH to a large extent.

To further investigate the inhibitory mechanism of WSC on yeast fermentation, we used the genome-wide, non-essential gene deletion mutant collection of *S*. *cerevisiae* to identify the chemical genomic profile of SynH + WSC versus ACSH. Sensitive and resistant gene mutants reveal the metabolic pathways that were affected by WSC, provide information for microbe genetic engineering [35], and confirm the observed variations between different hydrolysates and fermentation media (e.g. ACSH, SynH control media) [10,14]. Fig 6A shows the chemical genetic profile of WSC. In the chemical genetic profiling, a resistant mutant has a positive interaction score on the Y-axis, whereas a negative score indicates a sensitive mutant. For example, a deletion of *EEB1* (encoding Acyl-coenzymeA: ethanol O-acyltransferase involved in fatty acid biosynthesis) conferred resistance to WSC. Other resistant mutants included gene deletions of *SSH4* and *VAM6* (both involved in vesicle trafficking, which is the movement of important biochemical signal molecules from synthesis-and-packaging locations in the Golgi body to specific 'release' locations on the inside of the plasma membrane of the secretory cell). These gene deletions could be points for genetic engineering to help the yeast strain overcome inhibitory effects of degradation compounds present in lignocellulosic hydrolysates.

**Fig 7:**
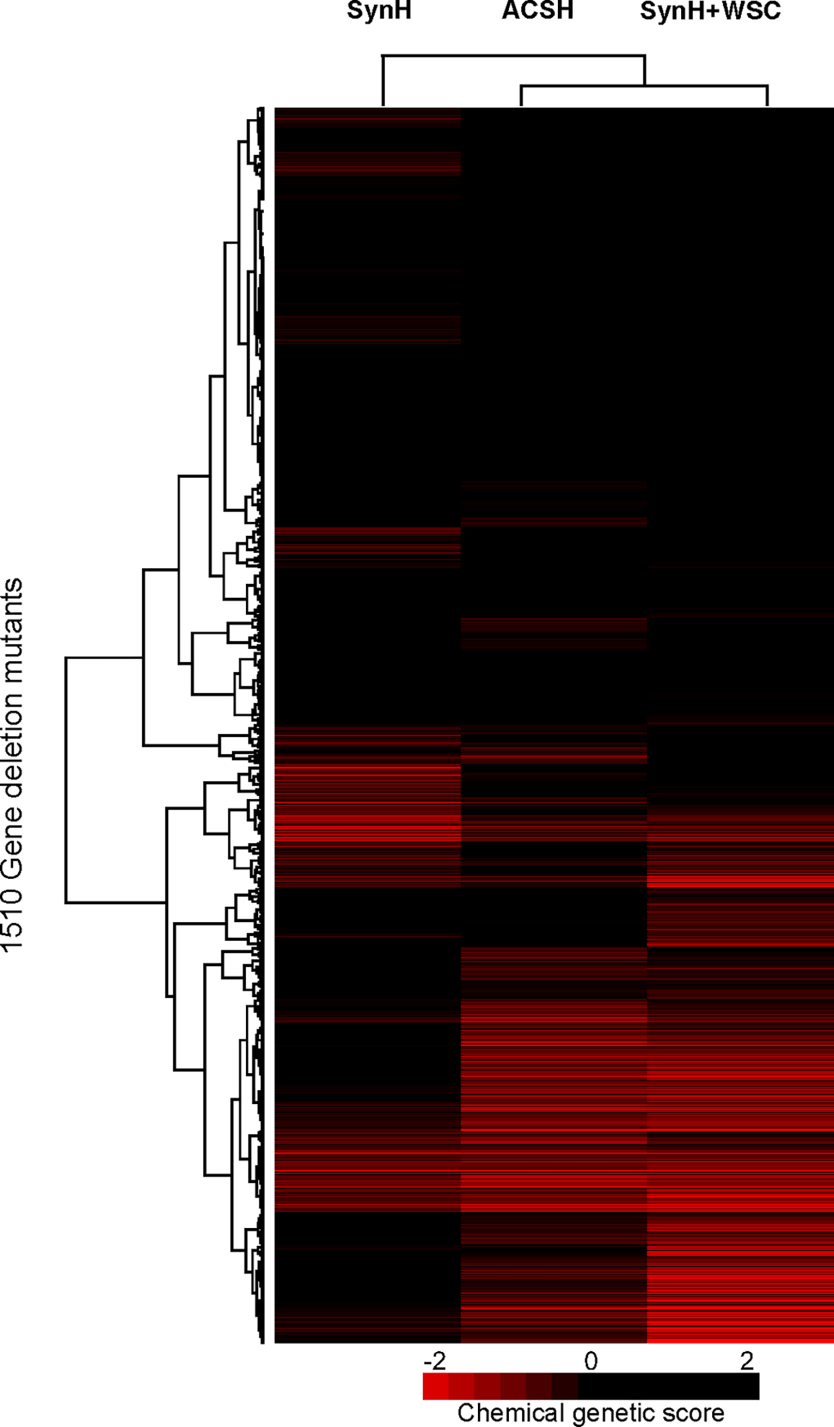
Correlation between chemical genomic profiles of SynH control media, ACSH and SynH+WSC. Chemical genomics is the study of chemical compound interactions with specific genes within an organism. This approach determined whether hydrolysate variability existed using a biological ‘‘sensor” (individual gene mutants) to create a genome-wide, biological ‘‘fingerprint” [14]. In this study, we combined chemical genomics profiling with SynH, therefore determined both hydrolysate variability and gene fingerprints. This is a high-throughput method to test different compounds for their inhibitory effects, which can be widely applied in fermentation study and media development.

## Conclusions

Lignocellulose-derived degradation products produced during thermochemical pretreatments are known to inhibit microbes during fermentation, and have been a major constraint in producing cost-effective lignocellulosic biofuels. In order to optimize the pretreatment processes for obtaining biomass and hydrolysates with higher digestibility and fermentability, respectively, and to engineer microbial strains for improved performance, it is critical to identify the major inhibitory compounds and better understand the mechanisms of inhibition that affect fermentative organisms. In this work, we used a synthetic hydrolysate (SynH) to study the inhibitory effects of water soluble compounds (WSC) isolated from the EA pretreatment crude lignin stream on yeast fermentation.

The natural lignin-derived compounds in WSC represent real inhibitory compounds that are present in ACSH. We found that the inhibitory effects of WSC increased at higher dosage levels, and SynH with 20 g/L WSC well mimicked real hydrolysates during yeast fermentation. Surprisingly, however, when the concentration of WSC reached 40 g/L, the inhibitory effects were reversed after 48 h, indicating that additional nutrients existed in WSC that manifested their effects at higher concentrations. To separate these effects and better understand this bimodal behaviour, ethyl acetate extraction was conducted to separate phenolic compounds from WSC. Major compounds identified in the organic phase of WSC included *p*-coumaroyl amide, feruloyl amide and coumaroyl glycerol. These and other nitrogenous phenolic compounds act as key inhibitors in microbial fermentation of ACSH.

On the other hand, oligosaccharides and lipids were also identified in the water phase components following ethyl acetate extraction. These compounds improved cell growth and ethanol production after 48 h, thereby explaining the bimodal effects of WSC. Finally, chemical genomic profiling of WSC was conducted on a yeast deletion library to identify responsive genes. This work proved that EA pretreatment improved hydrolysate fermentability in part by removing highly toxic lignin-derived nitrogenous and phenolic compounds.

The fundamental knowledge gained in this study can be applied to characterize a variety of inhibitory compounds, optimize fermentation process, and design pretreatment process conditions. This improved basic understanding will also help develop yeast strains that can better tolerate degradation compounds present in pretreated biomass hydrolysate.

## Supporting information

S1 Table Composition analysis of different fractions of WSC. (A). HPLC analysis of sugar and salts concentrations in WSC (g/L); (B). HPLC analysis of sugar and salts concentrations in water phase after ethyl acetate extraction (g/L); (C). Free amino acids analysis of WSC (μg/μL); (D). Mineral salts (heavy metal) analysis of WSC (ppm).
(PDF)

S2 Table Recipe of SynH being used for fermentation study. (A). Phosphate buffer, (NH_4_)_2_SO_4_ and salts (K/Na/Ca/Mg), amino acids, nucleic base, Vitamin; (B). Micro nutrients, FNSG salts, osmoprotectants, acetic and lactic degradation products, carbohydrates (carbon sources), Pyridines.
(PDF)

S1 Fig Methodology and mass balance of water extraction of lignin streams and ethyl acetate extraction of WSC. (PDF)

S2 Fig Fermentation media of SynH with different concentrations of WSC (unfiltered). SynH-1WSC represents 10 g/L WSC that were re-dissolved in the SynH fermentation media. From SynH-1WSC to SynH-4WSC, the concentrations of WSC increased from 10–40 g/L.
(TIFF)

## Abbreviations

WSC: Water-Soluble Components
EA: Extractive Ammonia
AFEX: Ammonia Fibre Expansion
ACSH: AFEX-Pretreated Corn Stover Hydrolysate
SynH: Synthetic Hydrolysates
SEC: Size Exclusion Chromatography
OD_600_: optical density or absorbance of a sample measured at a wavelength of 600 nm
LC-MS: Liquid Chromatography-Mass Spectrometry
GC-MS: Gas Chromatography-Mass Spectrometry
UHPLC: Ultra High Performance Liquid Chromatography
