# Supplementary material for: Water-soluble phenolic compounds produced from extractive ammonia pretreatment exerted binary inhibitory effects on yeast fermentation using synthetic hydrolysate
Source: PLoS One. 2018 Mar 15;13(3):e0194012. doi: 10.1371/journal.pone.0194012 (PMC5854342; doi:10.1371/journal.pone.0194012)
Supplement: S1 Table — (A). HPLC analysis of sugar and salts concentrations in WSC (g/L); (B). HPLC analysis of sugar and salts concentrations in water phase after ethyl acetate extraction (g/L); (C). Free amino acids analysis of WSC (μg/μL); (D). Mineral salts (heavy metal) analysis of WSC (ppm). (PDF) [file pone.0194012.s001.pdf]

S1 Table. Composition analysis of different fractions of WSC.

(A). HPLC analysis of sugar and salts concentrations in WSC (g/L);

HPLC (g/L)	Glucose	Xylose	Arabinose	Lactate	Glycerol	Formate	Acetate	Galactose
WSC	2.88	1.80	0.55	0.04	1.50	0.41	1.76	0.12

(B). HPLC analysis of sugar and salts concentrations in water phase after ethyl acetate extraction (g/L);

HPLC (g/L)	Glucose	Arabinose	Lactate	Glycerol	Galactose
Water phase	0.44	1.10	0.12	0.64	0.14

(C). Free amino acids analysis of WSC (μg/μL);

**Asp**	Asn	**Thr**	**Ser**	**Glu**
1.49E+02	0.00E+00	1.05E+02	1.66E+02	5.80E+01
**Ile**	**Leu**	**Tyr**	**Phe**	His
9.39E+01	9.00E+01	8.12E+01	9.79E+01	0.00E+00
**Gln**	**Pro**	**Gly**	**Ala**	Val
1.90E+02	3.36E+02	1.75E+02	6.05E+02	0.00E+00
**Lys**	Arg	Cystine	Methionine	Tryptophan
4.97E+01	0.00E+00	0.00E+00	0.00E+00	0.00E+00

(D). Mineral salts (heavy metal) analysis of WSC (ppm)

**P**	**K**	**Ca**	**Mg**	**S**	**Zn**	B	**Mn**	**Fe**
23.5	1013.7	20.4	110	105.6	0.73	< 0.04	1.4	0.1
**Cu**	**Al**	**Na**	Cd	**Co**	Cr	Mo	Ni	Pb
0.28	0.14	9.4	<0.008	0.01	<0.002	0.01	0.02	<0.04
**NH4-N**	**Total N**	**Cl**	**F**	NO2	Br	NO3	PO4	**SO4**
1624	5509	596.4	1148.5	<0.01	<0.01	<0.01	<0.02	348.7
