# Supplementary material for: Water-soluble phenolic compounds produced from extractive ammonia pretreatment exerted binary inhibitory effects on yeast fermentation using synthetic hydrolysate
Source: PLoS One. 2018 Mar 15;13(3):e0194012. doi: 10.1371/journal.pone.0194012 (PMC5854342; doi:10.1371/journal.pone.0194012)
Supplement: S2 Table — (A). Phosphate buffer, (NH4)2SO4 and salts (K/Na/Ca/Mg), amino acids, nucleic base, Vitamin; (B). Micro nutrients, FNSG salts, osmoprotectants, acetic and lactic degradation products, carbohydrates (carbon sources), Pyridines. (PDF) [file pone.0194012.s002.pdf]

S2 Table. Recipe of SynH being used for fermentation study.

(A). Phosphate buffer, $(NH_4)_2SO_4$ and salts (K/Na/Ca/Mg), amino acids, nucleic base, Vitamin;

Component	mM (Final Concn.)
KH_2PO_4	5.84
K_2HPO_4	11.15
$(NH_4)_2SO_4$	30
KCl	36.8
NaCl	1.3
$CaCl_2 \cdot 2H_2O$	5.5
$MgCl_2$. $6H_2O$	12.5
L-Alanine	1.172
L-Arginine.HCl	0.144
L-Asparagine	0.228
DL-Aspartic acid.K	0.594
L-Cysteine.HCl	0.050
L-glutamine	0.259
L-Glutamic acid.K	0.607
Glycine	0.378
L-Histidine	0.037
L-Isoleucine	0.262
L-Leucine	0.371
L-Lysine.HCl	0.175
L-Methionine	0.100
L-Phenylalanine	0.282
L-Proline	0.656
L-Serine	0.369
L-Threonine	0.310
L-Tryptophan	0.050
L-Valine	0.424
L-Tyrosine	0.202
Adenine	0.05
Cytosine	0.05
Uracil	0.05
Guanine	0.05
Thiamine HCl	0.0004
Calcium Pantothenate	0.003

(B). Micro nutrients, FNSG salts, osmoprotectants, acetic and lactic degradation products, carbohydrates (carbon sources), Pyridines

Component	mM (Final Concn.)
$ZnCl_2$	20
$MnCl_2 \cdot 4H_2O$	91
$CuCl_2 \cdot 2H_2O$	1.9
$CoCl_2 \cdot 6H_2O$	0.03
H_3BO_4	23.1
$(NH_3)_6Mo_7O_{24} \cdot 4H_2O$	0.31
$FeCl_3 \cdot 6H_2O$	20
Sodium formate	2.8
Sodium nitrate	1.1
Sodium succinate	0.5
Glycerol	4.1
Betaine.H2O	0.7
Choline Chloride	0.3
DL-Carnitine	0.3
Acetamide	80
Sodium acetate	32
L-lactatic acid (90%)	4
D-Mannose	1.2
L-Arabinose	20
D-Fructose	24
D-Galactose	2.9
D-Glucose	30 g/L (333 mM)
D(+)Xylose	30 g/L (200 mM)
Pyridoxine.HCl	2.14 µM
Nicotinic Acid	26.78 µM
Biotin	0.1 µM
Inositol	0.056 mM
