# Supplementary figures and images for: Water-soluble phenolic compounds produced from extractive ammonia pretreatment exerted binary inhibitory effects on yeast fermentation using synthetic hydrolysate
Source: PLoS One. 2018 Mar 15;13(3):e0194012. doi: 10.1371/journal.pone.0194012 (PMC5854342; doi:10.1371/journal.pone.0194012)

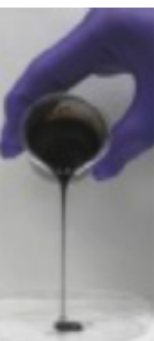

Water  
extraction

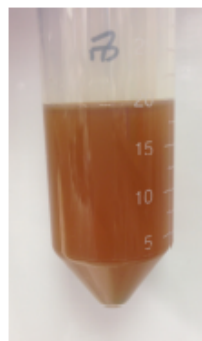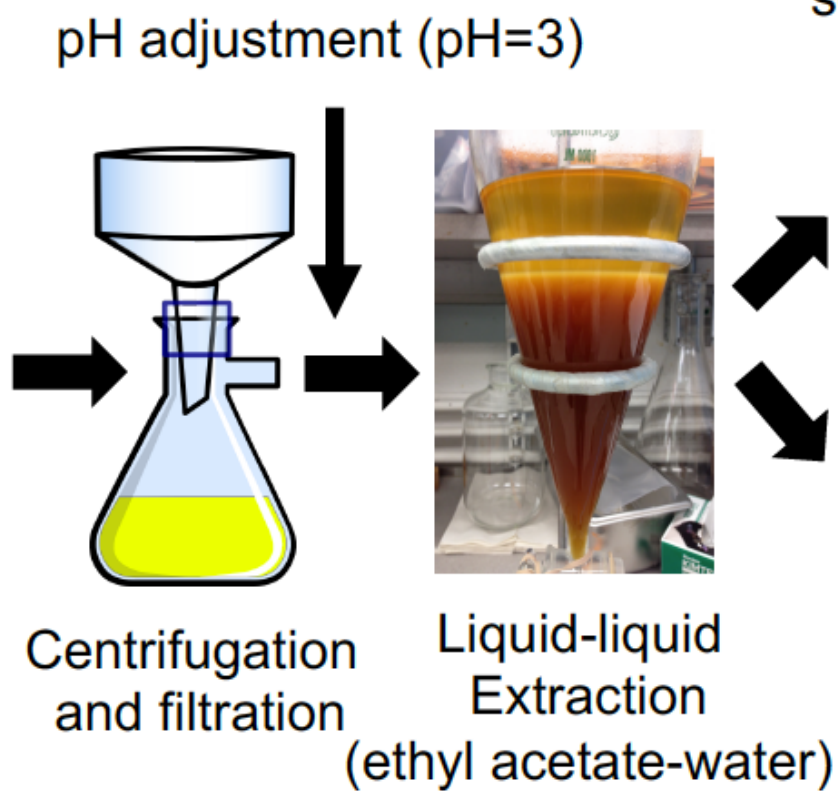

Phase  
separation

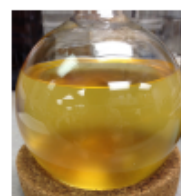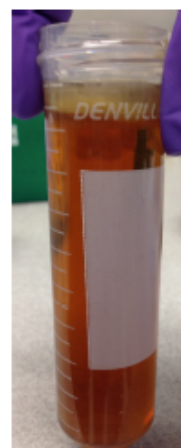

Rota-evaporation

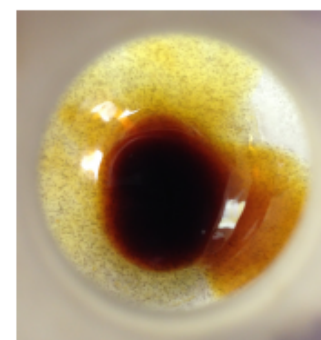

Phenolic  
compounds

Supplement: S1 Fig — (PDF) [file pone.0194012.s003.pdf]

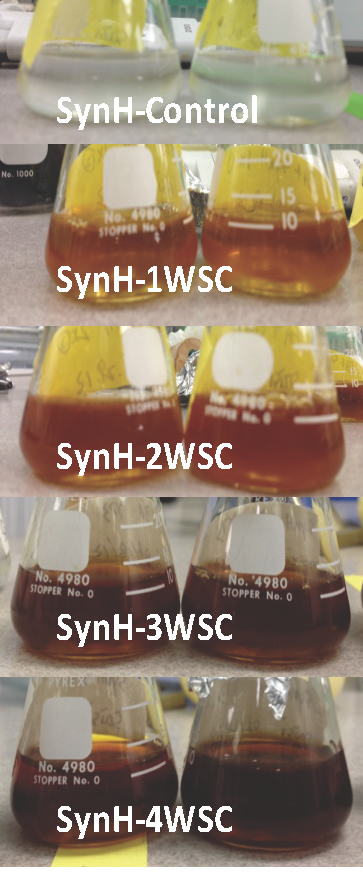

Supplement: S2 Fig — SynH-1WSC represents 10 g/L WSC that were re-dissolved in the SynH fermentation media. From SynH-1WSC to SynH-4WSC, the concentrations of WSC increased from 10–40 g/L. (TIFF) [file pone.0194012.s004.tiff]
